# Compensation Method for Die Shift Caused by Flow Drag Force in Wafer-Level Molding Process

**DOI:** 10.3390/mi7060095

**Published:** 2016-05-24

**Authors:** Simo Yeon, Jeanho Park, Hye-Jin Lee

**Affiliations:** Korea Institute of Industrial Technology, 143 Hanggaulro, Sangnok-gu, Ansan-si, Gyeonggi-do 15588, Korea; simo@kitech.re.kr (S.Y.); jeanho@kitech.re.kr (J.P.)

**Keywords:** wafer-level molding, compression molding, die shift, flow drag force, compensation method

## Abstract

Wafer-level packaging (WLP) is a next-generation semiconductor packaging technology that is important for realizing high-performance and ultra-thin semiconductor devices. However, the molding process, which is a part of the WLP process, has various problems such as a high defect rate and low predictability. Among the various defect factors, the die shift primarily determines the quality of the final product; therefore, predicting the die shift is necessary to achieve high-yield production in WLP. In this study, the die shift caused by the flow drag force of the epoxy molding compound (EMC) is evaluated from the die shift of a debonded molding wafer. Experimental and analytical methods were employed to evaluate the die shift occurring during each stage of the molding process and that resulting from the geometrical changes after the debonding process. The die shift caused by the EMC flow drag force is evaluated from the data on die movements due to thermal contraction/expansion and warpage. The relationship between the die shift and variation in the die gap is determined through regression analysis in order to predict the die shift due to the flow drag force. The results can be used for die realignment by predicting and compensating for the die shift.

## 1. Introduction

The development of semiconductor manufacturing technology for high-performance electronic products is actively pursued, and the degree of integration is undergoing a paradigm shift from two-dimensional (2D) packaging to three-dimensional (3D) packaging technology. Wafer-level packaging (WLP) technology is used for next-generation semiconductor packaging, and it implements high-performance and ultra-thin semiconductors in high-performance electronic products. WLP technology is studied as a core technology that will enable manufacturing cost savings and outstanding packaging performance, particularly in terms of electrical and thermal reliability. WLP technology possesses the advantages of fan-out packaging expandability and input/output increases, as the package bump extends to the external region of the silicon chip while allowing for WLP. This technology has the following characteristics [1,2,3,4,5,6,7,8,9].

1. WLP is achieved by implementing a 3D package, and it enables performance enhancement through a high pin count, an extension of existing silicon technologies, a reduction in device size, and a reduction in wiring delay.
2. Depending on the possibility of flexible process combinations, system semiconductors can be implemented.
3. WLP is an emerging packaging process technology that allows for high-performance semiconductor production and reductions in manufacturing cost.

To implement WLP packaging, it is necessary to resolve the problem of alignment between fabrication processes because a much finer pitch interconnection and a much higher pin count than those achieved with the multi-lithograph process, which is currently the most widely used packaging process, are required. Among all the processes, the WLP molding process has the highest defect rate, and it is thus a difficult process to predict. The WLP process embeds an epoxy molding compound (EMC)-packaged chip to form a molded wafer.

**Figure 1:**
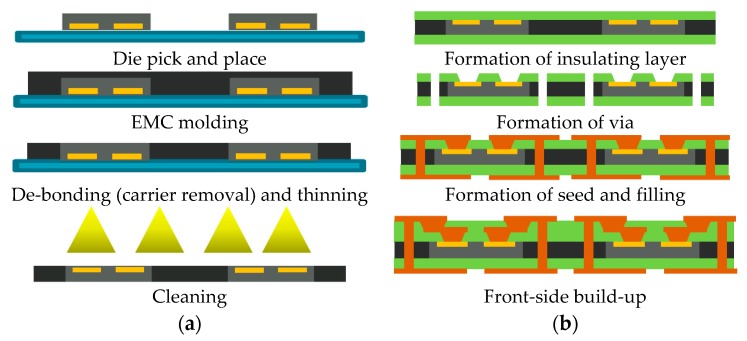
Wafer-level packaging process: (**a**) molding process; (**b**) redistribution layer process.

Die shift is a process defect that occurs during the molding process, in which the embedded die moves from its original position. Die shift is one of the most significant defects of the WLP molding process, and it causes misalignment during the subsequent process, as shown in Figure 1. Consequently, the quality of the molded wafer determines the quality of the entire package in the WLP process. In other words, die alignments after the molding process, followed by the re-distributed layer (RDL) process, are critical factors affecting the manufacturing yield of a package. The maintenance of the die alignment during the molding process is key for high-yield production with WLP.

Khong *et al.* [9] proposed a 2D numerical analysis method for the WLP molding process and carried out numerical analysis studies showing that die shift is caused by the pressure gradient in the flow filling stage. Ji *et al.* [10,11] proposed a numerical analysis method for the 3D WLP molding process and analyzed the flow resistance for the package model with numerous embedded dies to propose a guideline for molding tape selection. Sharma *et al.* [12] studied the effect of package specifications, operation temperature, and material on die shift and proposed a method to improve die shift by compensating for the measured die shift prior to the die-realignment stage. Ling and Bu *et al.* [13,14] proposed a numerical analysis method that predicts a die shift by considering the molding-tape deformation behavior during the molding process, and performed theoretical research for predicting the die shift caused by mechanical effects during molding.

The die shift caused during the WLP molding process results from the combined effects of process parameters including thermal contraction/expansion due to the change in temperature profile during the process, EMC flow drag force, and EMC curing shrinkage. Such die shifts are difficult to predict in advance, as the potential energy of the residual stress generated during the process is stabilized after the molding process, resulting in geometrical changes. The die shift problem must be overcome to minimize the setup time and cost of the initial process for commercial application. The number of experiments typically carried out for process setup is limited because of the time and cost. Accordingly, it is critical to determine the optimal design parameters to minimize the die shift phenomenon.

In this study, as a solution to this problem, a realignment method using die shift compensation data is investigated, and a guideline for package design is proposed. Moreover, to quantify the method’s performance, the die shifts caused during each stage of the molding process were evaluated, as well as the die movement caused by the geometrical changes and thermal expansion or contraction after the molding process.

## 2. Molding Process of Wafer-Level Packaging

### 2.1. Molding Process

**Figure 2:**
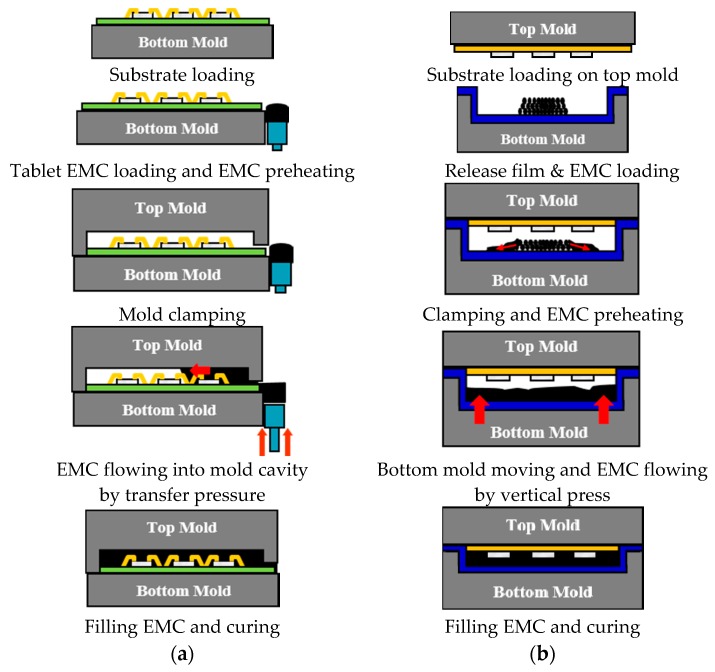
Comparison between semiconductor packaging molding processes: (**a**) transfer molding; (**b**) compression molding.

In semiconductor packaging processes, the molding process seals the packaged semiconductor chip using EMC in order to protect it from physical and chemical impacts, as well as from external humidity and contaminants. In general, the transfer molding method, which is currently the most widely employed semiconductor packaging molding process, is being replaced with compression molding as the preferred WLP molding process. This is due to the restrictions caused by residual stress and the lack of packing caused by the high flow length and low mold cap thickness over a large forming area. Figure 2 compares the two molding processes. A molded wafer is formed through a pressing process at low shear rates to minimize the flow of EMC, as shown in Figure 2b. Therefore, the compression molding process is being actively investigated for WLP molding. In this study, the compression molding method is investigated to evaluate the die shift value caused by the EMC flow drag force and to design the optimal package with minimum die shift.

### 2.2. Wafer-Level Compression Molding System

**Figure 3:**
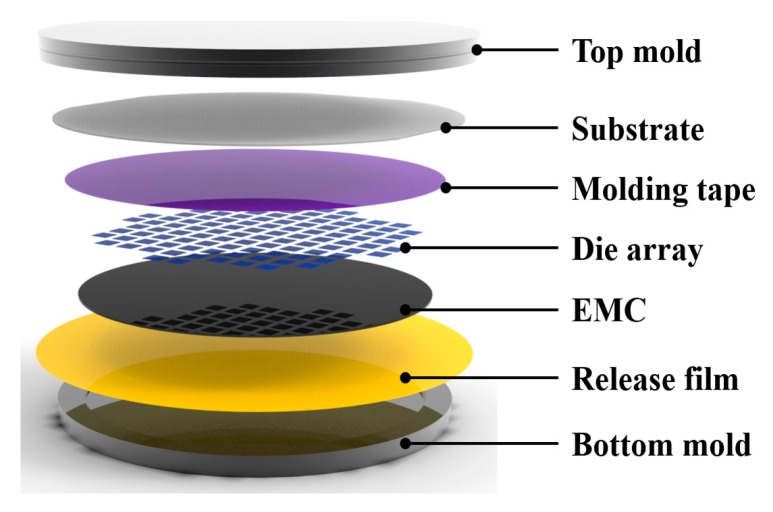
Components of compression molding.

**Figure 4:**
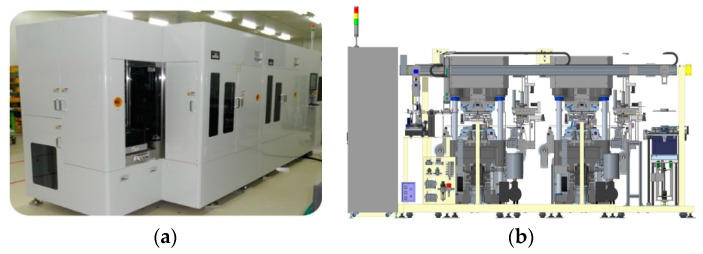
Detailed composition of the compression molding system: (**a**) wafer-level compression molding system; (**b**) system configuration.

WLP molding involves various materials in each step. As shown in Figure 3, WLP molding is composed of upper and lower molds, a substrate with package dies bonded using a molding tape, EMC, and a release film used to separate the formed EMC from the lower mold. In order to perform the molding process, the WLP compression molding system is required to satisfy a variety of criteria such as a high load, flatness of the product, and uniform temperature. In this study, we developed a WLP compression molding system by applying a toggle pressing method for performing the molding process. The wafer-level compression molding system is composed of a press, a compression mold, a thermal release film, and control modules. The detailed composition of the system is shown in Figure 4 and listed as follows:

- Mechanical press module: the maximum loading capacity is 100 tons, and the pressing mechanism is toggle-type
- Compression mold module: upper and lower molds are controlled at different temperatures
- Auto supply and recovery module of the release film
- Wafer loading and unloading module
- Post-curing and cooling module
- Automatic process control module

### 2.3. Wafer-Level Compression Mold Module

**Figure 5:**
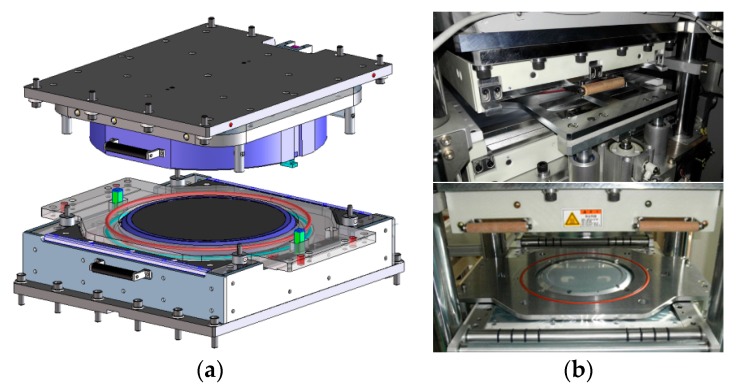
Compression molding die set: (**a**) design of the molding die set; (**b**) manufactured molding die set.

A compression mold module has been designed to remove air bubbles and gas from the EMC in a vacuum chamber, as shown in Figure 5. A cartridge heater was inserted into a heating plate having a grid shape to maintain a uniform temperature at the mold surface. To minimize temperature variation characteristics, individual control of the heater and a cooling line were applied to achieve uniform temperature on the mold surface. A further cooling line is designed to perform temperature profile control over the molding process.

## 3. Molding Process Experiment

**Figure 6:**
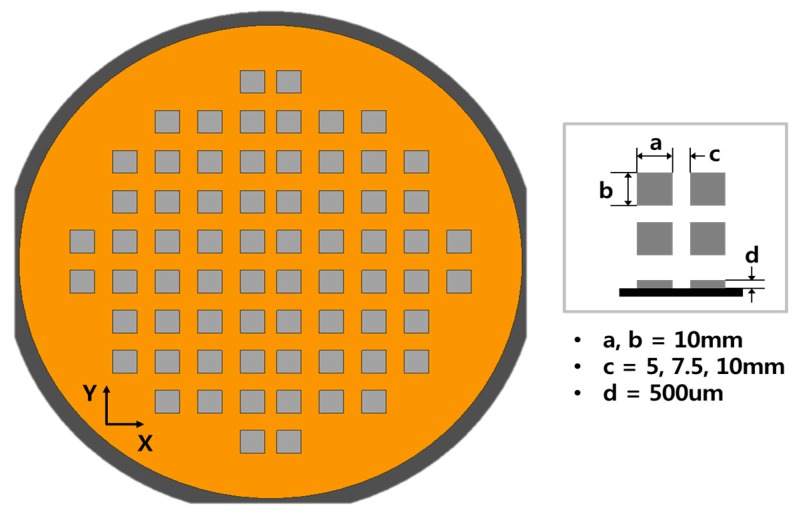
Design specifications of the wafer-level package (a: *X*-axis length of die; b: *Y*-axis length of die; c: die gap (gap between adjacent dies); d: die thickness).

In this study, an 8 in (approximately 200 mm) WLP molding process was investigated, in which the dimensions of the die used in the package are 10 × 10 mm^2^, the die thickness is 500 μm, and the die arrangement was a quarter-symmetric formation, as shown in Figure 6. The pick-and-place process of the die is performed using a die bonder (i-CubeII (YHP-2), Yamaha Motor Company, Shizuoka, Japan). The die gaps are designed as 5, 7.5, and 10 mm, which are 25% increments to 50% of the die size. A total of 68 dies are arranged for each process condition.

**Figure 7:**
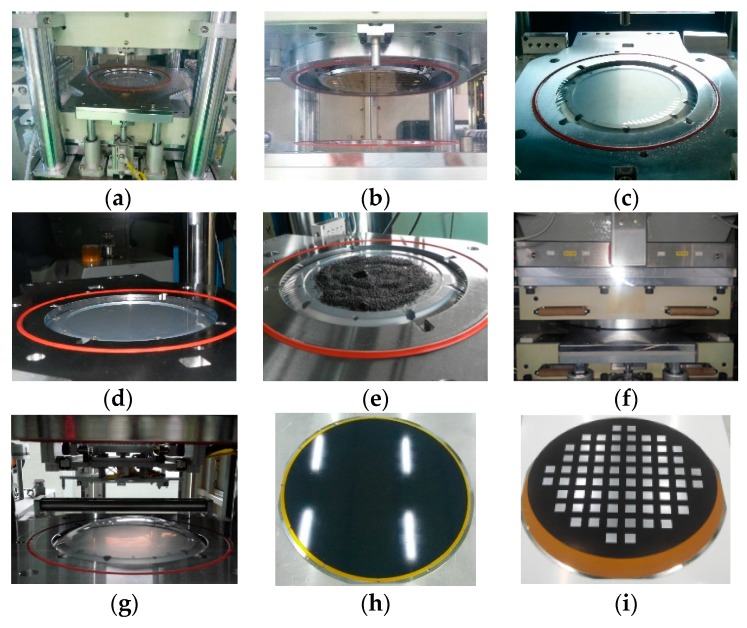
Experimental procedure of the compression molding process: (**a**) release film loading; (**b**) substrate loading; (**c**) release film clamp; (**d**) release film suction; (**e**) EMC loading; (**f**) mold pressing; (**g**) mold opening; (**h**) substrate unloading; (**i**) debonding.

Granular EMC is used for the WLP molding process, and Table 1 lists the material properties of granular EMC. The procedure of the WLP molding is described below and shown in Figure 7.

1. Place the release film on the lower mold maintained at a temperature of 170 °C.
2. Attach the die-arranged substrate to the upper mold using vacuum.
3. Perform release film binding using the release film support plate.
4. Attach the release film on the lower mold using vacuum.
5. Apply granular EMC on top of the release film.
6. Maintain the pressing force at 30 tons for 2 min until the forming process is completed.
7. Open the mold after the curing time.
8. Unload the molded wafer from the molding system, and perform the post-curing process for 1 h at 170 °C.
9. Debond the molded wafer on the substrate at 200 °C.

**Figure 8:**
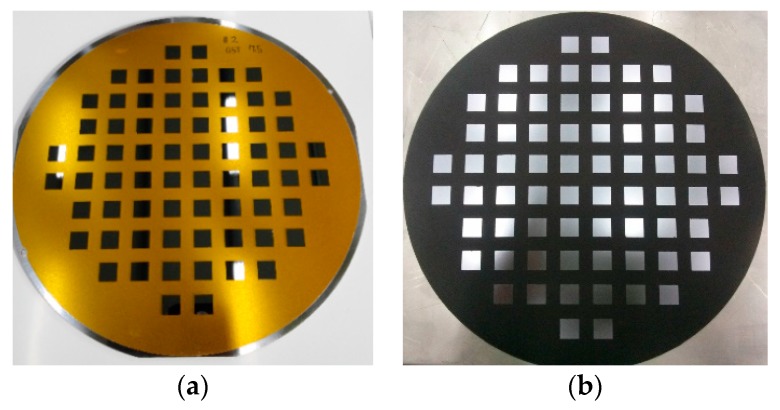
Specimen comparison: (**a**) before molding; (**b**) after debonding.

Die shift occurs because of complex factors such as warpage, thermal expansion/contraction, and EMC flow drag force during the molding process. To determine the change in the die position, the coordinates of the four die corners are measured before molding and after the debonding process by using a 3D profiler (PW-3300, PEMTRON Corp., Seoul, Korea), as shown in Figure 8. The center position of each die is calculated to assess the die movement. The geometrical shape change (warpage) of the molded wafer is evaluated by measuring the displacement of the die on the *Z*-axis after debonding. The die movements due to the thermal expansion/contraction of the substrate and the curing contraction of EMC are evaluated through numerical calculations. Table 2 lists the properties of the related materials.

## 4. Experimental and Analytical Results

**Figure 9:**
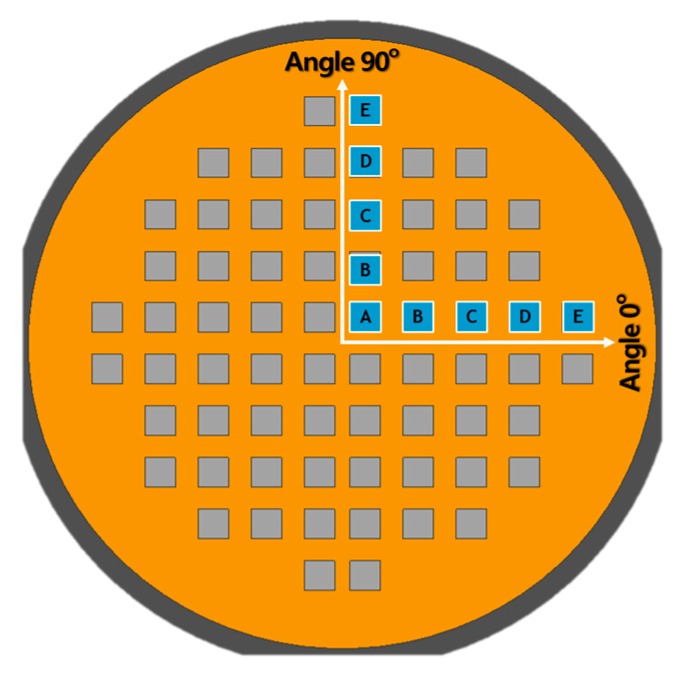
Definition of directions and nomenclature of measuring dies.

**Figure 10:**
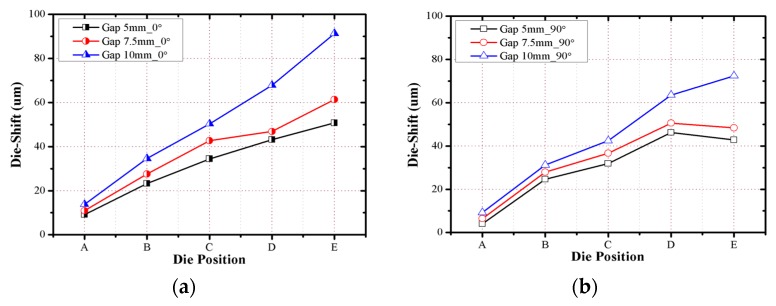
Die shift measurement results after the debonding process: (**a**) 0° direction; (**b**) 90° direction.

The die shift values measured after the debonding process are shown in Figure 9 and Figure 10 for the 0° and 90° directions. For both directions, the dies moved away from the center of the molded wafer, and the die shift values increased depending on the die gap increase. When the package die gap was less than 50% of the die size, the effect of the die gap on the die shift was low, but when the package die gap was increased to 75% of the die size, the die shift rapidly increased by more than 49%. The effect of the die shift on the molded wafer increased as the location of the die was farther away in the radial direction, and the die shift was observed to have a linear relationship with the radial position of the arranged die.

**Figure 11:**
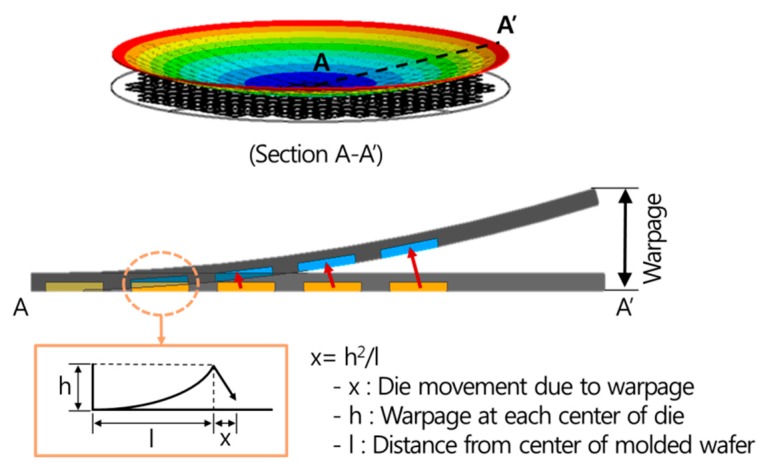
Evaluation of die shift caused by warpage.

The measured die shift, as shown in Figure 10, includes die shifts produced by various causes, including the thermal expansion of the substrate during the molding process, EMC flow drag force, thermal contraction and warpage after debonding, and contraction due to EMC curing. Therefore, it is important to distinguish die shifts according to their causes. The measured die shift positions before molding and after the debonding process show that the die movement is towards the center direction because of the warpage. Thus, trigonometry is utilized to evaluate the die movement quantitatively. The formula *x* = *h*^2^/*l* determining the warpage shown in Figure 11 represents the die movement caused by warpage occurring in the interval between the dies. In this study, this is calculated at each interval and cumulatively evaluated from the center to the outside.

**Figure 12:**
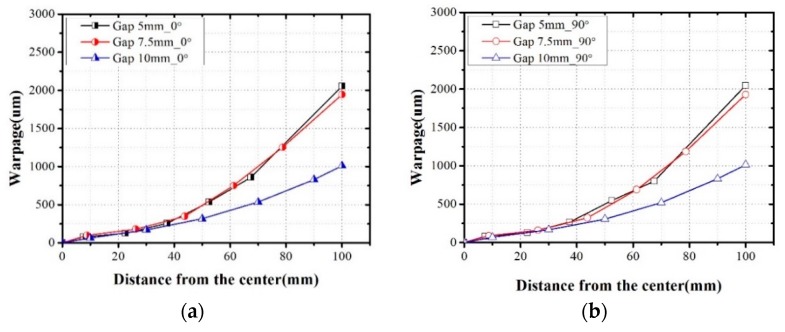
Warpage measurement results: (**a**) 0° direction; (**b**) 90° direction.

**Figure 13:**
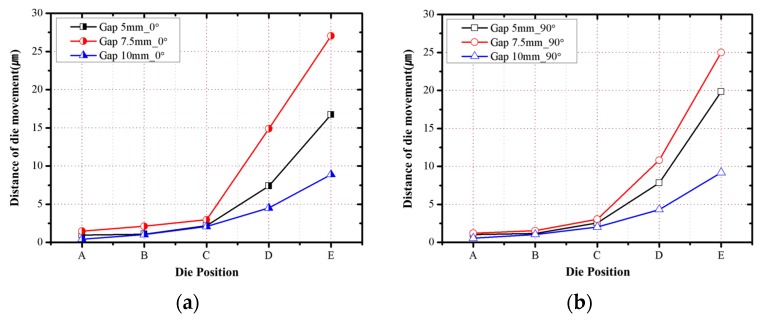
Evaluated die movement caused by warpage: (**a**) 0° direction; (**b**) 90° direction.

Figure 12 shows the warpage measurement results for the debonded wafer, and Figure 13 shows the evaluated die movement caused by warpage. The warpage increases as the die gap decreases, and it distinctly increases on the outside of the package, where the package die arrangement is uneven. The maximum warpage of the 5 mm case is greater by approximately 200% and 5% compared to those of the 10 mm case and 7.5 mm case, respectively. Therefore, the minimum die shift occurred in the case of the 10 mm die gap. The maximum die shift value of the 7.5 mm case is greater by approximately 25% than that of the 5 mm case. These results are attributed to the fact that the die position of the 7.5 mm case is farther from the center of the molded wafer and the maximum warpage is greater than in the 5 mm case; the last die positions of the 5 and 7.5 mm cases are 67.5 and 78.75 mm, respectively. The maximum warpage of the 7.5 mm case is greater by 39.2% (0°) and 22.1% (90°) compared to that of the 5 mm case.

**Figure 14:**
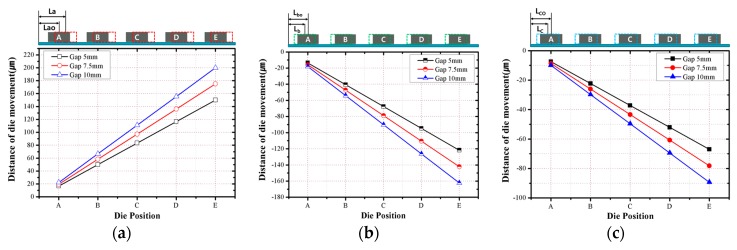
Evaluated die movement caused by thermal conditions: (**a**) thermal expansion during the molding process; (**b**) thermal contraction during the EMC curing process; (**c**) thermal contraction after the debonding process.

**Figure 15:**
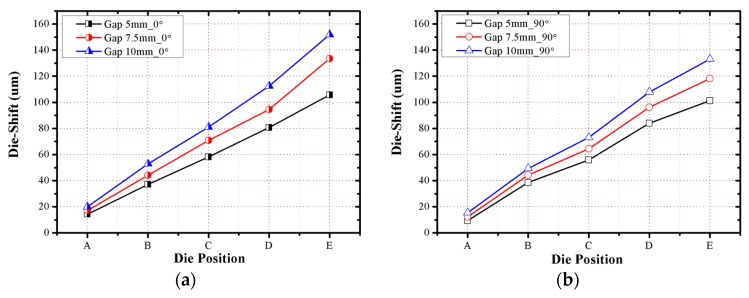
Die shift caused by EMC flow drag force: (**a**) 0° direction; (**b**) 90° direction.

**Figure 16:**
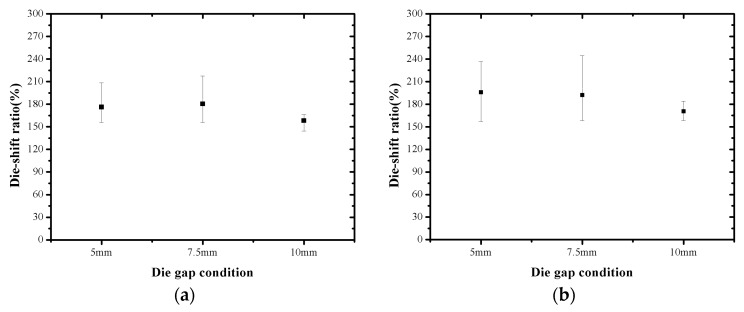
Ratio of die shift caused by flow drag force to that of the debonded wafer: (**a**) 0° direction; (**b**) 90° direction; (die-shift ratio = die shift caused by flow drag force/measured die shift of debonded wafer).

Die shift is also caused by temperature changes during the molding process. There are three factors affecting such die movement. First, as the temperature increases from room temperature (20 °C) to the molding process temperature (170 °C), die movement occurs because of the thermal expansion of the substrate, which has the bonded dies. Second, during the EMC curing process, die movement occurs because of the thermal contraction of the EMC. It is assumed that linear shrinkage occurs because die movement is evaluated in the section with low thickness. Finally, as the temperature of the molded wafer decreases from the debonding temperature (200 °C) to room temperature, die movement occurs because of the thermal contraction of the molded wafer. The position of the die is continuously modified in accordance with the temperature change profile. The values of the die position with respect to the center of the molded wafer are expressed by Equations (1)–(3), which are based on the original die position, coefficients of thermal expansion (CTEs) of the substrate or EMC, and temperature. Meanwhile, it was assumed that the die movement occurs linearly with the thermal expansion and contraction. By using the above equations, the relationship between the die position and die movement can be plotted, as shown in Figure 14. The die movement due to warpage and temperature changes compensates for the total die shift value. The analysis result indicates that the die movements due to the thermal contraction/expansion, EMC curing, and warpage occur in the direction towards the center. The die shift caused by the EMC flow drag force is evaluated to compensate for the measured die shift after the debonding process. The results are shown in Figure 15. To predict the die shift that will be generated by EMC flow drag force, the ratio of the die shift caused by the EMC flow drag force to the measured die shift after the debonding process is analyzed. As shown in Figure 16, the ratios in the cases of 5 mm and 7.5 mm die gaps are similar, but the ratio in the case of the 10 mm die gap is significantly less than those for the other two cases. This is because the warpage in the 10 mm case is less than that in the other cases; the maximum warpage in the 10 mm case is approximately half of that of the other two cases.

(1) $$L_{a}=L_{a0}\;\times \left(1+\;|{\text{CTE}}_{\text{substrate}}-{\text{CTE}}_{\text{silicon}}|\times 10^{-6}\;\times \Delta T\right)$$

(2) $$L_{b}=L_{b0}\;\times \left(1-\left(S_{\text{EMC}}\;\times 0.01\right)\right)$$

(3) $$L_{c}=L_{c0}\;\times \left(1-\;|{\text{CTE}}_{\text{EMC}}-{\text{CTE}}_{\text{silicon}}|\times 10^{-6}\;\times \Delta T\right)$$

(*L_a_*: length of thermal expansion; *L_b_*: length of curing shrinkage, *L_c_*: length of thermal contraction).

In order to intuitively predict and reflect the die shift due to the flow drag force during the molding process in the package design step, the relationship between the die shift and variation in the die gap must be determined. The die-realignment factor is determined through regression analysis, as expressed in Equation (4), and on this basis, the position-compensation relationship according to the die position in the package arrangement is derived, as expressed in Equation (5). The shape and arrangement of dies are symmetric, but the die shift data of the *X* and *Y* directions are different because of the asymmetrical shape of the substrate, as shown in Figure 6. Therefore, compensation equations are derived differently using the data shown in Figure 15. The basic die shift position is derived as the first terms on the right-hand sides of Equation (5) by using the dimensions of a die, and the compensated position of the other dies is derived using a realignment factor and the sequence number of a die. The above results can be used for die realignment by predicting the die shift in advance and compensating for it in the die-realignment process.

(4) $$\begin{array}{c} RF_{x}=2.06\times c+12.238\\ RF_{y}=1.27\;\times c+17.230 \end{array}$$

(*RF_x_, RF_y_*: realignment factor).

(5) $$\begin{array}{c} X_{com}=\left(2.29\times \left(\left(\;a+c\right)÷2\right)-2.968\right)+RF_{x}\;\times \left(N_{x}-1\right)\\ Y_{com}=\left(2.45\;\times \left(\left(\;a+c\right)÷2\right)-9.111\right)+RF_{y}\;\times \left(N_{y}-1\right) \end{array}$$

(*N_x_*, *N_y_*: die number from the molded wafer center).

## 5. Conclusions

In this study, the effect of die shift is investigated with respect to the variation of the die gap in the wafer-level molding process. The results can be used to compensate for the die shift caused by the flow drag force from the molded wafer. This compensation method can be applied through realignment in the package design step. The conclusions of this study are as follows.

1. The die shift increased with the die gap, and the die moved away from the center in the radial direction.
2. The warpage increases as the die gap decreases, and it distinctly increases on the outside of the package, where the package die arrangement is uneven.
3. The effect of warpage on the die shift increases suddenly for the dies arranged on the outside of a package.
4. The die shift caused by EMC flow drag force is evaluated from the data on die movements due to thermal contraction/expansion and warpage.
5. The relationship between the die shift and variation in the die gap is determined through regression analysis in order to intuitively predict and reflect the die shift due to flow drag force.
6. The die-realignment factor is determined and the position-compensation relationship is derived according to the die position in the package arrangement.
7. These results can be used for die realignment by predicting the die shift in advance and compensating for it in the die-realignment process.

## Figures and Tables

**Table 1:** Material properties of epoxy molding compound (EMC).

Property	Value	Unit
Spiral flow	36	inch
Gelation time	30	s
Shrinkage (*S*_EMC_)	0.18	%
Glass transition temperature (*T*_g_)	145	°C
Viscosity	85.652	Pa·s
Density	2.00 × 10^−3^	g/mm^3^

**Table 2:** Coefficient of thermal expansion (CTE) of molding components.

Material	Value	Unit
EMC (below *T*_g_ 150 °C)	8	ppm/°C
EMC (above *T*_g_ 150 °C)	49	ppm/°C
Substrate	17.3	ppm/°C
Die (Silicon)	2.62	ppm/°C
